# Following the footsteps: Urbanisation of Wa Municipality and its synergism in risk accumulation, uncertainties and complexities in urban Ghana

**DOI:** 10.4102/jamba.v11i1.479

**Published:** 2019-01-17

**Authors:** Martin Oteng-Ababio, George Owusu, Divine M. Asafo

**Affiliations:** 1 Department of Geography and Resource Development, University of Ghana, Ghana; 2 Department of Urban Studies and Planning, University of Sheffield, United Kingdom

## Abstract

Global demographic characteristics have witnessed a significant shift with more than half of the world’s population crossing the rural–urban threshold in 2008. In Ghana, the 2010 census report revealed 50.9% urban population. While the many benefits of organised and efficient cities are well understood, it must be recognised that rapid, often unplanned urbanisation brings risk of profound social instability, risk to critical infrastructure, potential water crises and the potential for devastating spread of disease. These risks can only be further exacerbated as this unprecedented transition from rural to urban areas continues. This also means stakes are high for public and private interventions to ensure that urbanisation reinforces rather than retards prosperity. In spite of these past experiences, urban governance policies in emerging smaller cities are frequently ambivalent and piecemeal, exhibiting similar negative tendencies, a development that has received less academic attention. This study adopted multiple research techniques and the data were generated through a structured questionnaire survey, personal interviews and discussions. Based on our conviction that the development trajectory of any city hinges on the quality of its physical foundation, we seek to fill the knowledge gap using the Wa Municipality, the least urbanised but one of the fastest urbanising cities in Ghana today, as a case study. The results reveal emerging tendencies that indicate that Wa appears to be following in the footsteps of its predecessors – experiencing an inefficient potable water supply system and chronic sanitation situation, making diarrhoea one of many challenges for residents. It is ultimately suggested that a collaborative partnership with all key stakeholders is a better option to reap the potential for urbanisation to strengthen economic growth and development.

## Introduction

The growth of the population in cities dates back in time, but the phenomena seem to have peaked in the last decades of the 20th century and the first decades of the 21st century (Grant 2009, 2015; Mitlin & Satterthwaite 2013). Today, about 40% of Africa’s population live in cities and towns, with the urban population growing 14-fold from 32 million in 1950 to over 450 million in 2014 and expected to triple to over 1.3 billion by 2050 (Turok 2016). Associated with the rapid urbanisation is an equal increase in the rate of the physical footprint of these cities, which per the current trends could be more than half in 2050 (Angel 2016; Turok 2016). Such burgeoning urban population and physical expansion, if not properly managed, poses daunting challenges for communities, the ecosystem and local authorities (Cartwright 2015; Turok 2016; UN-Habitat 2014). This is particularly the case when the urbanisation rate is happening at much lower levels of gross domestic product, per capita and economic growth than has occurred elsewhere (Freire, Lall & Leipziger 2014; World Bank 2010). Put differently, as cities in Africa grow, their inability to ensure effective spatial planning, create jobs, provide basic social services and infrastructure results in the development of slums, the use of unsafe water, poor sanitation and dwelling in substandard structures (Bull-Kamanga et al. 2003; Pelling & Wisner 2009). An opening remark of the *State of the World’s Cities* report in 2008 affirmed that ‘cities are the materialisation of humanity’s noblest ideas, ambitions and aspirations, but when not planned or governed properly, can be the repository of society’s ills’ (UN-Habitat 2008:X). This is evident in the case of Lagos, Kampala and other cities that are characterised by recurring flood disasters (Christie & Hanlon 2001; ActionAid 2006 cited in Pelling & Wisner 2009).

Ghana is not an exception to these urban dynamics and the associated challenges as most of its large cities including Accra and Kumasi are paying the avoidable price for unguided urbanisation. Studies in Ghana so far (see Grant 2009; Owusu & Oteng-Ababio 2015) indicate that economic growth and job creation have lagged well behind urban population growth, creating a plethora of challenges and having wide-ranging implications for city authorities to provide urban infrastructure and public services. Oteng-Ababio (2014) and Songsore (2008) highlight the ill-planned nature of Accra’s urbanisation processes and how it has exposed some residents, especially those in the low-income communities, to incessant cholera outbreaks, floods and fire hazards. In most instances, government response has been swift but ambivalent and rather costly. This normally leads to piecemeal investments and reflects authority’s reservations about the consequences of urbanisation and, perhaps, the lack of appropriate institutions and framework to project appropriate responses (see Grant 2009; Myers 2011). This growth and accompanying risk accumulation processes are microcosms of Accra and Kumasi (see Oteng-Ababio 2014; Songsore et al. 2014). The National Urban Policy (2012), for example, was intended to guide the urbanisation processes in Ghana with the potential for urban growth to support economic development by harnessing the advantages only recently being recognised (MLGRD 2012). In spite of these glaring opportunities, emerging cities such as Wa, the Upper West regional capital, are exhibiting identical tendencies. Moreover, because Ghana only crossed the 50% urbanisation threshold in 2010, it was expected that there is an opportunity for emerging cities to do things differently and not to ‘follow in the footsteps’ of their predecessors.

The main purpose of this article is to consider the pathways for environmental risk accumulation in the rapidly urbanising and increasingly expanding Wa Municipality. The paper unpacks the current situation by examining residents’ access to basic infrastructural services such as potable water, solid waste management (SWM), sanitation and how the absence of these services generates uncertainties and health complexities in Wa. We see these services as public goods and as unlikely to emerge spontaneously through the operation of market forces and private initiatives alone. It calls for all-inclusive governance and collective action.

### Complexities, uncertainties and development in cities

Associated with urban advantage is the widely held view of cities characterised by dysfunction and microeconomic consequences such as poor housing, inadequate urban services, inadequate access to infrastructure and limited capacity to cope with impending disasters (Cardona et al. 2012; Dickson et al. 2012). From that background, this paper adopts the framework of Dickson et al. and the concept of the urban penalty to explain the complexities and uncertainties associated with the growth of Wa. According to Dickson et al. (2012), understanding urban disaster risk is based on the interplay of three principal pillars: institutions, hazards and socio-economic characteristics of a city’s population. Thus, urban uncertainties reveal the outcome of complex development policies, existing vulnerabilities and exposure of the population to hazard events (Cyr 2005; Dickson et al. 2012; Wisner et al. 2003). According to Pelling and Wisner (2009) and Dodman et al. (2013), the interplay results from unguided urbanisation, inefficient governance, ineffective planning and uneven provision of social services including potable water and proper solid waste disposal methods. This assertion further resonates with Cardona et al. (2012), who noted that urban disaster risk is largely attributed to vulnerable conditions caused by interaction between social, political and environmental processes. In furtherance to unpacking the processes of these risks, this article adopts the concept of the urban penalty to discuss the implications of these complexities and highlight the spatial dynamics of these uncertainties characterising the growth of Wa.

The urban penalty primarily theorises that increasing morbidity of an urban population is because of cumulatively poor environmental and social conditions in cities experiencing rapid growth (Dodman et al. 2013; Freudenberg, Galea and Vlahov. 2005). Commenting on the possible evidence of the urban penalty in the sub-Saharan regions, Gould (1998:179) mentioned that:

… without urgent and substantial commitment to urban improvement – in the public domain and in the domestic domain, and by international donors and agencies as well as by national governments – there really might then be a serious threat of an ‘urban penalty’ emerging in Africa within the next decade, and particularly for the rapidly growing mass of the urban poor. (cited in Harpham 2009:112)

This highlights the current situation in large cities, including emerging cities such as Wa, where the urban penalty is evident because of the concentration of poor people and their exposure to poor physical and social conditions resulting from urban governance (Freudenberg et al. 2005:1; UNISDR 2014). The urban poor, who are mostly characterised by their informal settlements and slums, poor housing conditions, poor access to potable water sources, poor sanitation and drainage facilities among others, are the most affected population paying these penalties (McGranahan 2007). This tendency and trend of development in cities are what Pelling and Wisner (2009) describe as the ‘tale of two cities’, where the poor pay a penalty by wallowing in unplanned areas underserved with basic social services while the affluent enjoy 24/7 access to social services with well-planned spatial layouts. Dickson et al. (2012) affirmed this point, revealing that the urban poor are the most vulnerable to disaster risks on account of overcrowding in housing structures served by inadequate basic services in the face of unsustainable income and weak social capital. The implication and evidence of this penalty are increasing disease epidemics such as cholera, diarrhoea, malaria and typhoid among the urban poor (McGranahan 2007; Songsore et al. 2009).

Conclusively, these challenges unwrap decades of development efforts and reverse the gains of poverty reduction (UNISDR 2008). Bendimerad (2003) corroborated this viewpoint, stating that disaster risk damages infrastructure, destroys the environment, decreases economic potential, disrupts small businesses and reduces human capital as a result of deaths and injuries. Therefore, understanding the fundamental factors defining uncertainties and complexities in cities will ensure sustainable urban development and promote resilient cities, an objective captured in Sustainable Development Goal 11 and the Sendai Framework for Disaster Risk Reduction (UNDESA 2015; UNISDR 2015).

#### Study area: Profile and growth of Wa

**FIGURE 1:**
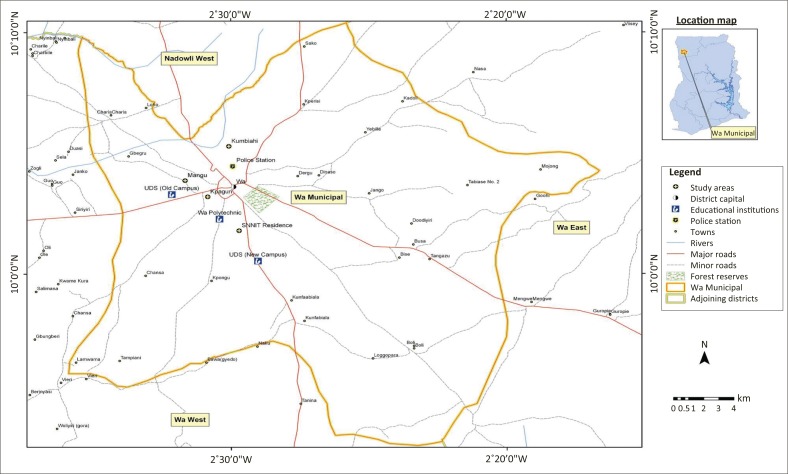
Map of Wa Municipal area.

Wa is the last of the ten regional capitals created in Ghana in 1983 (GSS 2002a) and located in the north-western part of Ghana (see Figure 1). Until 2010, the agriculture sector employed a majority of Wa’s population, but in recent times the service sector has taken over, employing 51.3% of the population as against 30.2% employed by the agriculture sector and 18.4% in the industrial sector, a situation depicting a typical municipal economy in Ghana (UNDP 2011; GSS 2013a). The shift in the economic structure (from agriculture to services) further reveals the pattern and trend of many Ghanaian cities, whose urban processes are built on population increase without any influence of manufacturing.

**FIGURE 2:**
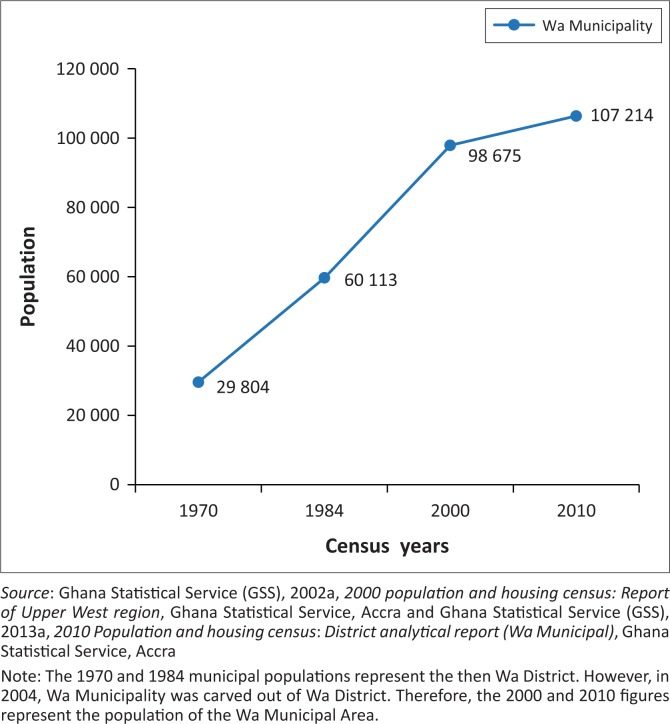
A graph showing the rapid urbanisation of Wa Municipality.

Several intercensal reports (see Figure 2) on Wa reveal a significant growth in the area’s population. The population of Wa increased from 29 804 in 1970 to 60 113 in 1984. It further increased to 98 675 in 2000 and subsequently to 107 214 in 2010 (GSS 2002b, 2013a). The 2010 population census report reveals that the municipality recorded the highest concentration (66.3%) of urban dwellers as against the regional and national figures of 16.3% and 50.9%, respectively (GSS 2013a, 2013c). Indicatively, although Wa is the least urbanised in the hierarchy of regional capitals in Ghana, it is indeed experiencing rapid growth.

The genesis of urbanism in Wa has its root in the 15th century, when Wa became the headquarters of the Wala State, engaging in major trade activities with then-Islamic Mande and Hausa traders (Songsore 1985). Large-scale urbanisation, therefore, commenced in 1983, when the Upper West region was carved out of the then Upper Region with Wa as the regional capital, an attempt to bridge the development gap between the area and the rest of the country (GSS 2002a). Subsequently, in 2004, the decentralisation policy of 1988 adopted by Ghana upgraded Wa to municipal status through Legislative Instrument 1800 (WMA 2013). More recently, the establishment of the University for Development Studies (Wa Campus) in 2002 and the Wa Polytechnic in 1999 (Peprah 2013) has increased the population of Wa. In addition, the completion of major trunk roads (the Wa–Kumasi trunk road, the Wa–Tamale trunk road) is observed to have further opened the Wa Municipality for easy movement of goods and services.

These factors, coupled with the municipality serving as an economic growth pole in the region, have attracted many tertiary students, civil and public servants into the municipality. The implication of this population growth is the increasing demand for basic services such as potable water, housing, sanitation and health facilities in the municipality (Ahmed & Dinye 2011; Peprah 2014). Moreover, the conversion of open spaces and pavement for commercial activities has resulted in haphazard development, leading to congestion and overcrowding of the urban space (Ahmed & Dinye 2011).

## Methods

Using Wa Municipality as a case to understand the processes of risks in emerging cities, the study used a mixed research method approach. This enabled the study to generate various datasets and provide an in-depth explanation for the complexities and uncertainties associated with the urbanisation processes of Wa (Tashakkori & Teddiem 2010 cited in Teye 2012). The quantitative method adopted the use of a questionnaire, which was administered to 200 respondents sampled through a four-level multistage sampling technique. The first stage involved the stratification of the municipality into high-class residences, low-class residences, middle-class residences and newly developed residences using the criteria of income levels and infrastructure availability, data that were obtained from the Town and Country Planning Department of Wa Municipal Assembly. The high-class residences are characterised by an affluent population residing in low-density and well-planned communities with access to social services including potable water, sanitation facilities and proper solid waste disposal systems. The other areas, especially the low-class residential areas and newly developing areas, are characterised by a high-density population with poor housing conditions and deteriorating social services. The second stage involved random selection of one community from each of the stratified areas. The study selected Social Security and National Insurance Trust residence to represent the high-income residence, Kumbiehe as the newly developed area, Kpaguri as the middle-income residence and Mangu as the low-income residence.

In the third stage, a simple random selection method was employed to select houses in the communities. In the Social Security and National Insurance Trust (SSNIT) residence, every third house was selected because of the linear nature of the settlement. However, because of the haphazard nature of settlements in the other three communities, settlements were clustered into four zones using landmarks and streets as boundaries and a simple random sampling technique was applied to select the houses. The last stage involved the selection of household heads who responded to the survey questions. This was necessary for the study to capture quality data based on respondents’ experiences of the changing socio-economic characteristics of the population in the municipality as well as the accumulating risks in the area. A proportional allocation method was adopted to ensure fair representation in each community. The population of these communities from which the samples were drawn was as follows: low-income residences, 3461; middle-income, 3014; high-income, 1128; and newly developed areas, 627. Based on this, 42% of respondents were sampled from low-income areas, 36.5% from middle-income residences, 13.7% from high-class areas and 8% from newly developed residences. This result discloses the population density of the municipality, where the low-income residents, for instance, have a high population density compared to the high-income areas. In addition, this feature also reveals the population distribution in most municipalities in Ghana where there is evidence of high-class residential areas with low population density and low-class residential areas with high population density. The questionnaire probed the socio-economic characteristics of residents and their daily practices of solid waste disposal, sanitation and access to potable water. The data were analysed using the descriptive statistics tool from the Statistical Package for the Social Science (SPSS) software, version 23.0 to generate tables, line graphs and bar graphs to represent participants’ responses.

Qualitative interviews were also conducted to complement the survey results. Semi-structured, open-ended questions were used to interview the key stakeholders. The discussions probed into various strategies adopted to ensure effective provision of basic social services and the associated challenges faced amidst the increasing population in the municipality. The seven key informants who were interviewed included two assembly members from Mangu and Kumbiehe, and officers of the Environmental Health Unit, the National Disaster Management Organisation, the Municipal Planning Office, Ghana Water Company and the Town and Country Planning Department, all of Wa Municipal Assembly. Additionally, two focus group discussions were conducted for landlords (nine participants) and tenants (nine participants) in the municipality. These landlords and tenants were randomly recruited during the survey process conducted in the low-income areas. The discussion focused on how landlords were responding to the rapid urbanisation and the challenges they were facing as key players in housing provision. Discussion with tenants in Mangu also examined the daily activities that were making them susceptible to disaster risks. Daily activities of residents involving access to water, sanitation and solid waste management were also observed. The discussions from the interviews were transcribed, coded and themed into several topics or ideas. However, the actual voices of the respondents are used in this study to augment and further provide meaning to support the quantitative data.

## Findings

### Infrastructure provision in Wa: Access to drinking water

The Ghana Water Company Limited (GWCL) by law is the institution responsible for water supply in all urban areas in Ghana. However, increased population has resulted in the demand for potable water outpacing supply, hence the problem of inadequate piped water supply in the municipality. In a discussion on the sources of water supply by GWCL and the volume produced per day, an official of the company revealed:

‘… the demand for water currently in Wa is 10 000 m³ per day but due to our limited resources and sources of water, we are making an average of 1200 m³ a day. We operate 16 boreholes and the yields of these boreholes are not all that good. This is the cause of our inability to meet the demands of the people.’ (GWCL-Wa, 18 February 2014)

**FIGURE 3:**
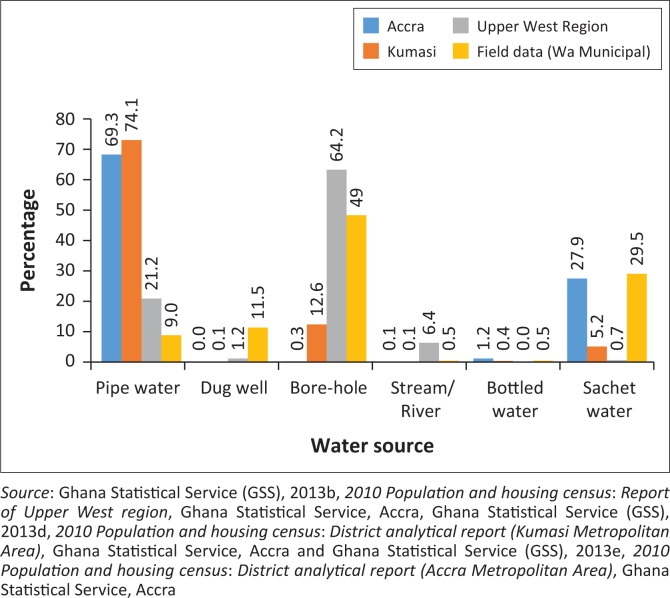
Sources of water used for drinking.

Figure 3 presents the various sources of drinking water used in the communities. The findings show that only 9% of respondents have access to piped water against the regional urban coverage of 21.2% and national coverage of 64.4% (GSS 2013c). This deficiency in piped water supply in Wa re-echos the situation in most large metropolitan areas, where water supply to some communities is compromised. A case in point is where 69.3% of the 1 665 086 population in Accra and 71.1% of the 1 730 249 population in Kumasi have access to piped water. The results resonate with the urban penalty paradigm, which, as already indicated, posits that cities concentrate poor people and expose them to unhealthy physical and social environments, as well as, in this case, lack of supply of potable water (Freudenberg et al. 2005).

Further analysis of the data revealed that 49% of the respondents access boreholes as their source of drinking water. This observation is in tandem with the regional average of 64.2% of the population of the region (Upper West) relying on boreholes (Ghana Statistical Service 2013b). During the key informant interviews, an official from GWCL explained that the reliance on boreholes in the municipality and beyond is a result of the unavailability of surface water sources. Significantly, Wa’s case manifests the proliferation of boreholes drilled by individuals (mostly affluent) who mechanise and operate them for commercial purposes. An opinion leader at Mangu remarked:

‘… the pressure on water is so huge that it has caused many private people to drill and mechanise boreholes and they are making a lot of money from it. Nonetheless, the fact still remains that tapping groundwater isn’t a solution. In some communities, there is no point in digging wells as the underground water is all brackish.’ (Assemblyman for Mangu, 2014)

Apart from the use of boreholes, another major source of drinking water identified by our respondents was sachet water, accounting for 29.5%, a finding that resonates with the 27.9% recorded in Accra (Ghana Statistical Service 2013e). Known colloquially as ‘pure water’, sachet water refers to 500-mL sealed plastic sleeves of purified drinking water that have become ubiquitous in most urban areas as a result of generally high quality and low cost (US$0.03–$0.06) for both producers and consumers.

Additionally, findings of the study show that the problem of inadequate water supply is unevenly distributed. The study revealed that residents in the low-income area (Mangu) are victimised as there was no piped infrastructure at all in the community during the study, compelling residents to depend solely on boreholes (both public and private ones) with a myriad of challenges as alluded to by the assemblyman of the community. The situation contrasts with that of high-class neighbourhoods, which apart from well-laid pipes are also privileged with reservoirs for water storage and private boreholes that serve residences in times of low water supply from the GWCL. The Assemblyman for Mangu (a low-income community) further lamented:

‘… in Mangu, we do not have a single pipeline that runs through the community. It’s the boreholes that we have, few public ones and many others by individuals who are capitalising on the lack of such an invaluable resource and are charging any amount but still, we must go for them!’ (Assemblyman for Mangu, 6 February 2014)

This situation reflects the common circumstances of water provision in large cities where the affluent are well served with piped water at the expense of the poor, who struggle with lack of adequate services.

Our findings further revealed that the few residents who could not afford piped or borehole water resorted to other improvised sources such as dug wells, dams, streams and rivers. Although our study did not conduct any physical examination of these sources, earlier studies have highlighted that their severe contamination with human and animal waste, fallen debris and cross-contamination by farm animals have plunged residents into health risks, increasing residents’ susceptibility to water-related diseases such as diarrhoea (Songsore et al. 2014; Stoler 2013). McGranaham (2007) also indicated that an unreliable supply of potable water is the leading cause of increasing water-related diseases especially in urban areas (cited in Oteng-Ababio 2014).

#### Solid waste management

**FIGURE 4:**
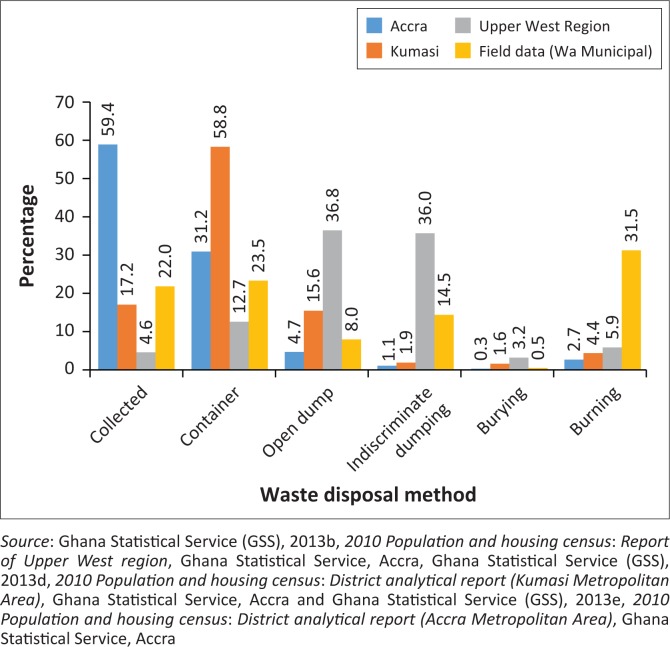
Method of solid waste disposal in Wa Municipal Assembly.

**FIGURE 5:**
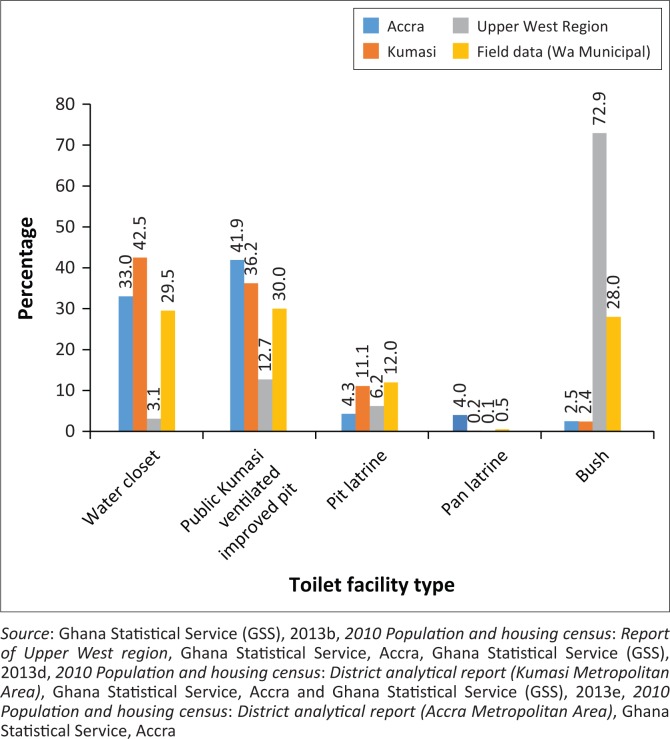
Type of toilet facility used by respondents.

SWM among residents in Wa Municipality takes various forms and practices. The study uncovered that (see Figure 4) less than half of the population (45.5%) have their waste collected through door-to-door collection and central communal container (CCC) systems, respectively. Per data collected from the Environmental Health Unit of the Wa Municipal Assembly, the city has only 40 communal containers. Meanwhile, the findings revealed that most residents are unable to patronise the door-to-door waste disposal method, which costs Gh⊄30.00 for registration and a monthly commitment fee of Gh⊄10.00 at the time of the study. As a result, open drains, uncompleted houses and open spaces in Wa have become a receptacle for plastics and empty water sachets. This condition depicts a lagging situation in SWM in Wa compared to other large cities such as Accra and Kumasi, where a significant number of people (see Figure 5) patronise CCC and also have their solid wastes collected. The discouraging SWM practices in Wa confirm earlier statistics, which show that only 4.6% and 12.7% of the population have their solid waste collected through the door-to-door and CCC systems, respectively (GSS 2013a).

The ineffective application of the door-to-door and CCC systems of SWM in cities has compelled some residents (31.5%) to engage in burning of their solid wastes. Others also dispose of their waste indiscriminately and in open public dumps in Wa (14.5% and 8%, respectively). The realities of SWM practices in Wa follow a similar trend in large metropolitan areas where waste remains uncollected and is dumped on undeveloped lands and in drains. Relatively, the case of Accra and Kumasi does not differ (see Figure 5) as several residents engage in burning and dumping of solid wastes in open spaces.

Similarly, the challenge of SWM in Wa is also skewed as people in high-class residences engage in proper practices. Our results show that most residents in SSNIT (77.8%) use door-to-door collection methods for waste disposal while the rest use the CCC that is located within the community (see Table 1). However, the low-income communities remain underserved with solid waste infrastructure. As witnessed in Mangu, more than half of the respondents (51%) burn their solid waste, while 23.8% and 14.3% engage in indiscriminate dumping and open dumping, respectively. Commenting on the sanitation challenges in Mangu, an assemblyman noted:

‘… solid waste management is affecting Mangu a lot! An area with an estimated population around 7000 (electoral area) has no single community container! no single community container! And this has been a worry to me. I complain a lot to the Assembly but still, you don’t know what they are doing.’ (Assemblyman for Mangu, 2014)

**TABLE 1:** Cross tabulation of location of respondents and method of solid waste disposal.

Location	Improved		Unimproved			
	Door-to-door collection	Public dump (container)	Public dump (open)	Indiscriminate dumping	Burying	Burning
Kpaguri	17.8	50.7	2.7	9.6	1.4	17.8
Mangu	6.0	4.8	14.3	23.8	0.0	51.2
Kumbiehe	31.2	6.2	6.2	12.5	0.0	43.8
Social Security and National Insurance Trust	77.8	22.2	0.0	0.0	0.0	0.0
**Total**	**22.0**	**23.5**	**8.0**	**14.5**	**0.5**	**31.5**

**FIGURE 6:**
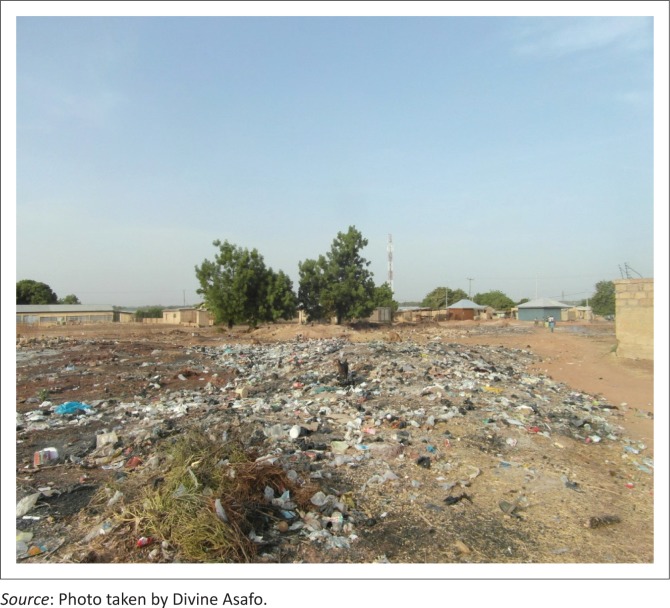
An illegitimate open dump site in Kpaguri, Wa.

Figure 6 shows an illegitimate open dump site in Wa. According to the Assemblyman for Kpaguri, dumping in this area is usually done at night or at dawn when people are asleep. This spatial disparity in solid waste disposal practices reflects the situation in the largest cities in Ghana where the high-income areas patronise door-to-door collection services while the low-income areas struggle with a few communal containers, which are always left spilling over and uncollected. A daunting implication for the health of the population, therefore, includes the contracting of upper respiratory tract infections from smoke emitted from burning waste. Coupled with this challenge is the potential flood risk threatening most residents as a result of the choking of open drains with solid waste, a situation identified as a major contributing factor to flooding occurrences in Ghanaian cities (Oppong 2011).

### Sanitation practices

The challenge of access to improved sanitation in Wa Municipality is low. The study found that (see Figure 5) less than a third of respondents (29.5%) use water closets. This reflects the general situation in the region where only 3.1% of the population use water closets. The low sanitation coverage undoubtedly accounts for the 72.9% of the region’s population engaging in open defecation. Consequently, the lack of sanitation facilities in most communities has resulted in 30% of the respondents using public toilets (Water Closet/Kumasi Ventilated Improved Pit [WC/KVIP]). The limited number of public KVIPs (46) in the municipality has compelled a significant number of respondents (27%) to engage in open defecation, literally known as the ‘free range’. A major cause of this environmental challenge in Wa is the complexity of increasing demand for housing, inadequate planning, inefficient enforcement of building laws and indiscipline, which has given landlords the leverage to build houses without sanitation facilities. The situation mirrors the dreadful sanitation in Accra and Kumasi, where less than half of the population use water closets. Thus, many residents rely on public toilet facilities while others use pit latrines and engage in open defecation.

Noticeably, just as for SWM practices, the burden of inadequate sanitation is unevenly distributed (see Table 2). Essentially, in the high-class areas (SSNIT residences), residents (100%) have water closets in their houses, while the remaining communities depend on public toilets, pit latrines and open defecation. In Mangu in particular, 47.6% of the residents engage in open defecation, creating indiscriminate human waste found in open spaces, bushes, uncompleted buildings and drains.

**TABLE 2:** Type of sanitation used by communities.

Location of respondents	Type of toilet facility (%)					
	Water closet	KVIP	Pit latrine	Pan latrine	Bush	Other
Kpaguri	16.4	47.9	19.2	0.0	16.4	0.0
Mangu	16.7	26.2	8.3	1.2	47.6	0.0
Kumbiehe	37.5	18.8	18.8	0.0	12.5	12.5
Social Security and National Insurance Trust	100.0	0.0	0.0	0.0	0.0	0.0
**Total**	**29.5**	**30.0**	**12.0**	**5.0**	**28.0**	**0.0**

KVIP, Kumasi ventilated improved pit.

During a focus group discussion, we identified that the high engagement in open defecation by the low-income residents was as a result of an attitudinal challenge on the part of tenants and landlords. A tenant from Mangu noted:

‘… some landlords in Wa have a problem with building latrines in their houses. They get their tenants before they put up all these things. They claim the toilet is expensive but I think they just don’t want it. Even those with the toilet at home, when it’s full, they don’t drain it. They leave it like that and it overflows. This is the reason why we engage in “free range”.’ (Focus Group Discussion, tenant from Mangu, February 2014)

Confirming the assertion made by the tenants, a landlord asserted:

‘… we do not have enough money to build toilets. We normally concentrate on where to sleep before thinking of where to defecate.’ (FGD; landlord from Mangu, February 2014)

Meanwhile, a major health implication of open defecation is the excruciating stench emanating from these areas, polluting the ambient air and the increasing breeding of houseflies, which carry pathogens from human waste and deposit them on cold and uncovered foods. Residents’ sources of water such as dams, rivers and dug wells are also threatened as a result of the washing of faecal matter into them during flash floods in the rainy season.

## Discussion

The study analyses the growth of Wa with respect to the provision of social services such as drinking water, SWM and sanitation. From the analysis, Wa, like most cities in Ghana, is experiencing rapid urbanisation (GSS 2013c). Consequently, the large and rapid influx of people into the municipality has not only created new demands for social services, such as health and education, but more importantly has placed greater strains on the physical infrastructure, including water and sanitation facilities (Ahmed & Dinye 2011; Peprah 2013). In the words of Songsore (2009), Wa is experiencing ‘demographic urbanisation’, fuelled by a natural increase in rural–urban migration rather than industrialisation, which induced urbanisation processes in developed countries (see also World Bank 2015). The city authorities therefore have the monumental task of redressing the limited and unequal distribution of basic services. According to our study, the provision of basic water supply and environmental sanitation is a growing priority in Wa today.

Urban scholars (see Cardona et al 2012; Cohen 2006; Pelling & Wisner 2009) have argued that the incapacity of city authorities to provide basic services for its increasing population is the cause of uncertainties and complexities in the cities of most developing countries. This situation has become the bane of authorities in large cities in Ghana with evidence of persistent flood risk, fire, cholera and diarrhoea epidemics (Oteng-Ababio 2014; Songsore et al. 2014). Subsequently, emerging cities and municipalities are equally experiencing these urban development processes and their associated challenges. As this case study has amply demonstrated, the case of Wa cannot be underestimated. Although the area is experiencing growth, municipal authorities lag behind in the provision of basic services such as piped water supply, sanitation and proper SWM practices.

Significantly, the study establishes that the use of other unapproved sources of water such as sachet water, dug wells and dams is the outcome of the low coverage of piped water in the municipality. However, it is important to stress that while sachet water, for example, has been responsible for improving water access in many water-stressed neighbourhoods, particularly low-income and slum communities, the discarded plastic sleeves have become a sanitation menace and a contemporary hot button issue in the city and beyond. Indeed, plastic sachet wrappers litter most streets in the country today and clog drains and gutters in the rainy season, increasing the likelihood of floods and leading to subsequent public exposure to untreated sewage and a melange of health risks. Reminiscent of the urban penalty paradigm, the urban poor become exposed to unhealthy physical and social environments. This finding resonates with a Ghana Statistical Service (2008) report, which revealed that insufficient potable water supply compels urban residents to access water from unapproved sources (cited in Nyarko & Hayward 2011).

Beyond the inadequate supply of potable water is the proliferation of private water vendors who produce and sell sachet water. Their efforts help to build the legitimacy of the local state and give local settlers leverage to obtain basic services such as water. A peculiar situation in Wa is the proliferation of mechanised boreholes, which are operated by the affluent to support the water system in the municipality. However, as the study revealed, many residents (especially the poor) are unable to patronise them, compelling them to access other unsafe water sources. Further, improper practices of sanitation and SWM such as open defecation, indiscriminate dumping, burning and dumping of solid waste in open drains are a common situation in Wa. This confirms a recent World Bank report, which notes that most of the urban population lacked access to proper waste disposal services (World Bank 2015), while Songsore et al. (2014) note additionally that most residents result to burning, burying and indiscriminate dumping as a result of the authorities’ inability to collect waste generated in the municipality. Similarly, the disparity in the distribution of basic services in the municipality has also been skewed towards high-income areas, leaving low-income residents underserved.

Crucially, the study finds ample proof of the existence of the urban penalty, especially visible among low-income communities in Wa Municipality and by extension urban areas across the country, where lack of safe drinking water, improper sanitation services and poor SWM practices continue to yield a substantial morbidity and mortality burden, much of which is related to urban environments and lifestyles taking their toll on people’s health. Despite the prevalence of these disadvantages in marginalised and deprived communities, improved services, for the most part, were also much faster in affluent neighbourhoods within the municipality. The study suggests that both the existence of the ‘urban penalty’ and ‘safe havens’ in the same city can best be understood in terms of the nature of towns and of urban life, society and government. As noted by Oteng-Ababio (2014), high-class residents in cities have fully furnished and effectively managed basic services while their counterparts in low-income areas suffer from inadequacies in basic service provision. The finding resounds with the study by Songsore et al. (2014) in Accra, which also revealed that informal settlements such as Agbogbloshie, Old Fadama and other indigenous low-class residential areas such as Jamestown resort to beaches, open spaces and bushes to defecate as a result of the lack of toilet facilities in their homes.

As a matter of fact, the inadequate toilet facilities in low-class residences in most urban areas have resulted in some people defecating in polythene bags, otherwise called ‘flying toilets’, and throwing them in bushes or into a dump site (see Oteng-Ababio 2014; Songsore et al. 2009, 2014; World Bank 2015). Consequently, the existence of most urban dwellers especially the poor is threatened by the inadequacy of basic services. Indeed, data collected from the Municipal Health Directorate in Wa confirmed a high level of relationship between poor environmental conditions in Wa Municipality and the high prevalence of diarrhoea cases. For instance, the reported number of diarrhoea cases (in Out Patient Department) increased from 2181 cases in 2008 to 12 828 in 2013. Additionally, the municipality has been experiencing flash floods in recent times as the open drains have become receptacles of waste materials. Clearly, per the study, the urban poor in Wa are being penalised with the negative outcomes of unguided urbanisation in the municipality. The municipal authorities often have neither the financial resources nor the administrative capacity to extend services rapidly to the poorest neighbourhoods. Increasing access to urban services is likely to become a crucial problem and its solutions will require a combination of innovative and creative approaches. Hopefully, looming crises from poor environmental services will provide authorities with the insight they need to develop resilient programmes for the cities.

## Conclusion

Undeniably, urban population growth in Ghana and its associated challenges is a realistic experience. Hence, much confirmation can be summoned from the persistent flood and disease epidemic occurrences in metropolitan areas including Accra, Kumasi and Tamale. Significant among these disasters is the recording of a cumulative total of 28 955 cholera cases in Ghana in 2014 with 70% cases recorded in Greater Accra (WHO 2014). According to UN-HABITAT (2013), these threats are capable of degrading the health of the urban population, especially the poor, who face the penalty of deprived environmental conditions.

As a matter of fact, the growth in emerging cities such as Wa, exhibiting the same trends, patterns and cumulative challenges as those occurring in large metropolitan areas, poses a serious threat to the agenda of building sustainable urban development and resilient cities. Therefore, attempts to mitigate and possibly prevent disaster risks can no longer be intensified and limited to only large cities but also must be enacted in emerging cities to avoid the annihilation of development prospects and placing untold pressure on the country’s budget. Also, losing focus on these urban development trends can thwart the plan for meeting the Sustainable Development Goals, especially if the increasing number of urban poor in small towns are not furnished with basic services.

In conclusion, this article suggests an all-inclusive collaboration with the private sector in providing infrastructure and services for the municipality. Specifically, public–private partnership strategies should be extensively implemented to engage private entities to provide specific services such as potable water, sanitation, housing and proper solid waste disposal systems for the population. Lastly, the study calls for the intensive education of residents on the need to ensure environmental sustainability and to reduce their susceptibility to disaster risks. Thus, this article believes engaging these measures will establish more urban advantages other than penalties and reduce the vulnerability and exposure of urban populations to disaster risks.
